# Hydatid cyst of thigh diagnosed on ultrasonography - a rare case report

**Published:** 2012-06-18

**Authors:** A Gupta, RP Singal, S Gupta, R Singal

**Affiliations:** * Department of Anatomy, Adesh Institute of Medical Science and Research, Bathinda, Punjab, India; ** D. Ortho, F.C.G.P, M.R.S.H. (London); Department of Orthopedics, Adesh Institute of Medical Science and Research, Bathinda, Punjab, India; *** Department of Radiology, Maharishi Markandeshwar Institute of Medical Sciences and Research, Mullana, Distt-Ambala, Haryana, India; **** "Dr Kundan Lal" Hospital, Ahmedgarh, Distt-Sangrur, Punjab, India

**Keywords:** subcutaneous, hydatid cyst, thigh, surgery

## Abstract

Hydatid disease is a parasitic disease that is endemic in many parts of the world, especially in South America, the Middle East, Africa, Australia, and the Mediterranean region, including Turkey. This article presents an unusual case of subcutaneous hydatid cyst in the right medial side of the thigh. According to our world literature search, few cases are reported. This site of localization is very rarely diagnosed on ultrasonography.

## Introduction

More than 90% of the hydatid cysts occur in the liver, lungs, or both [**1**]. Peripheral organ hydatidosis is much less common, as few embryos can escape the capillary filtrating systems of the liver and lung. Primary hydatid disease of the skeletal muscle is rare and is present in approximately 3% of the patients [**2**]. Theoretically, the muscle is inhospitable for echinococcal infestations because of its contractility and high level of lactic acid [**3**]. Preoperative diagnosis and avoidance of diagnostic biopsy or aspiration is crucial in preventing local recurrence, cystic infection, and anaphylactic shock.

## Case report

A 38-year-old man reported with a swelling in the medial aspect of the right thigh for the past 10 months. The swelling was gradually increasing in size. There was no history of trauma or fever. The history of pain was present in the thigh. On the examination, a non-tender swelling felt in the subcutaneous area of the right medial aspect of the thigh. Swelling was firm in consistency, which was free from the underlying structures. Blood tests were within normal limits. 

**Fig. 1 a,b:**
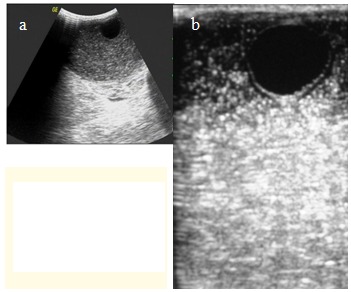
Ultrasonography revealed daughter cysts in the right thigh

Ultrasonography (USG) revealed a cystic lesion of a size of approximately 5 x 7 cm in the subcutaneous area of the right thigh (**Fig. 1 a,b**).

There was evidence of multiple daughter cysts within the cystic lesion along with few floating membranes. Probable diagnosis of subcutaneous hydatid cyst was made. The rest of the organs were normal. On the computed tomography (CT) of the thigh, there was a well-defined cystic mass in the antero-medial subcutaneous area of the left thigh. Bones and muscles of the rest of the thigh were normal. The patient underwent surgery with a probable diagnosis of hydatid cyst. 

On exploration, a cystic mass was revealed in the subcutaneous area of the right thigh. On cut section of the cyst, its color was white. The skin was closed primarily after thorough wash. On histopathological examination, diagnosis was made as hydatid cyst. The postoperative period was uneventful and the patient was discharged on albendazole at a dose of 400 mg. In follow-up of 8 months, the patient was asymptomatic without any recurrence.

## Discussion

Muscular hydatid cysts may be primary, but may also occur secondarily when cysts spread from other areas, either spontaneously or after previous operations for hydatidosis, in other regions of the body. The exact incidence of musculoskeletal hydatidosis is not clear. According to various authors, the incidence of musculoskeletal echinococcosis including involvement of subcutaneous tissue is around 1 – 5.4% amongst all the cases of hydatid disease [**4,5**]. 

The majority of the infestations are caused by echinococcus granulosus and multilocularis [**5,6**]. More than 90% of the hydatid cysts occur in the liver, lungs, or both, but subcutaneous area is supposed to be an unfavorable site for infestation because of its high lactic acid concentration. There are a few reports about primary subcutaneous hydatidosis in the extremities [**7**]. Chevalier et al. have reported the incidence of subcutaneous hydatid cysts as being of approximately 2%, but some of their patients had hydatid cysts also in other organs [**7**]. The primary soft tissue involvement is very rare, causing a diagnostic challenge. We came across a rare case of subcutaneous hydatid that was diagnosed on USG and confirmed on histopathology. 

Preoperative diagnosis can be made radiologically on USG, computed tomography, or magnetic resonance imaging by the characteristic appearance of a unilocular or multilocular cyst with multiple daughter cysts as it was seen in our case, in USG. Surgery is the treatment of choice for hydatid cysts without any rupture. However, large cysts are drained intra-operatively, irrigated with a scolecidal agent such as the hypertonic saline, and then excised. A chance of an anaphylactic reaction can develop if the cyst ruptures with a leakage of cyst contents [**8**]. Intraoperative irrigation of 0.5% cetrimide, 15% hypertonic saline and 0.5% silver nitrate solution, before the cyst opening, it may kill the daughter cysts and further reduces the risk of dissemination and anaphylactic reaction. The choice of treatment is surgery. The combination of adjunctive chemotherapy with antihelminthics is recommended to cover the risk of dissemination during the initial exploration. The medical treatment should precede and follow the surgical treatment.

## Conclusions

The purpose of this case report is to create a level of awareness, that in an area where the hydatid disease is endemic, patients coming with a soft tissue cystic swelling, the possibility of hydatid cyst should be kept in differential diagnosis. A hydatid cyst is rarely seen in thigh and its diagnosis is rarely made on USG.
